# Effect of curvature on wetting and dewetting of proboscises of butterflies and moths

**DOI:** 10.1098/rsos.171241

**Published:** 2018-01-17

**Authors:** Chengqi Zhang, Charles E. Beard, Peter H. Adler, Konstantin G. Kornev

**Affiliations:** 1 Department of Materials Science and Engineering, Clemson University, Clemson, SC 29634, USA; 2 Department of Plant and Environmental Sciences, Clemson University, Clemson, SC 29634, USA

**Keywords:** wetting, capillarity, Lepidoptera, feeding, fibre

## Abstract

Proboscises of butterflies are modelled as elliptical hollow fibres that can be bent into coils. The behaviour of coating films on such complex fibres is investigated to explain the remarkable ability of these insects to control liquid collection after dipping the proboscis into a flower or pressing and mopping it over a food source. By using a thin-film approximation with the air–liquid interface positioned almost parallel to the fibre surface, capillary pressure was estimated from the profile of the fibre surfaces supporting the films. The film is always unstable and the proboscis shape and movements have adaptive value in collecting fluid: coiling and bending of proboscises of butterflies and moths facilitate fluid collection. Some practical applications of this effect are discussed with regard to fibre engineering.

## 1. Introduction

Butterflies and moths (Lepidoptera) are opportunistic feeders, consuming a wide range of fluids, from thick, highly viscous liquids, such as honey, to thin, almost inviscid mineral water [1–4]. Lepidoptera, like all insects, struggle with capillary forces. John Haldane highlighted a uniqueness of lepidopterans and certain other insects: *‘*An insect going for a drink is in as great danger as a man leaning out over a precipice in search of food. If it once falls into the grip of the surface tension of the water—that is to say, gets wet—it is likely to remain so until it drowns. A few insects, such as water beetles, contrive to be unwettable; the majority keep well away from their drink by means of a long proboscis’ [5].

The lepidopteran proboscis is a highly flexible device capable of a wide range of actions, from coiling in the vertical plane to prehensile-like movements for acquiring liquids and pollen [1]. These movements can, for instance, alter the spacing of the interlegular gaps to regulate fluid entry into the food canal, enlarge the food canal of the narrowed apex to reduce the suction pressure required to bring fluids to the mouth and package the proboscis in a tight coil when not in use [1].

The chemical composition of the proboscis includes hydrophobic chitin in its cuticle, coupled with surface lipids and waxes [6–9]. These abundant materials are integrated in a manner allowing the insects to solve the dual challenge of acquiring fluids from diverse nutrient sources while maintaining a clean proboscis [10,11]. The roughness of the proboscis surface significantly changes the surface energy, decreasing it in the case of waxed hydrophobic patches and increasing it in the case of protein-rich hydrophilic patches [12–16].

Drinking from floral corollas, insects encounter nectar in the form of pools, droplets or films adhering to the corolla surface, often in limited quantities. The extent of insertion of the proboscis into the corolla influences the extent of the proboscis exposed to fluid [17–20]. However, the physical principles of nectar movement to the food canal are poorly understood.

Lepidoptera are often observed drinking mineral water from the soil by bending their proboscises into a J-configuration with the distal portion pressed to the ground [21–24]. The physical phenomena that facilitate feeding when the proboscis is bent are unknown [2,25–27]. Generally, in the bent or coiled proboscis, the series of legular plates are prone to open up the interlegular pores [17]. This effect can favour fluid uptake when the insect feeds, but it is unfavourable for maintaining moisture in the proboscis: when the proboscis is coiled, the interlegular pores could allow evaporation if they remain open. This structural arrangement would seem to contradict the terrestrial organism's need to minimize evaporation [7,27–29].

When the insect acquires a thin liquid film after each dip of the proboscis into a flower or other food source, the problem of delivery to the food canal is reduced to the analysis of the flow of this liquid film. Before formulating the fluid mechanics model for this process, an understanding of the driving forces causing the flow of thin films towards the food canal is required. We focus this study on the analysis of these forces.

We studied the capillary effect of a liquid film associated with a flexed or coiled proboscis to answer the question of how coiling and bending the proboscis might benefit the insect. An understanding of wetting phenomena of complexly shaped fibres is relevant not only to insect biology but also to materials engineering [12–14,30–36]. We examine the stability of liquid films on a hollow fibre, with elliptical cross section, coiled in a ring. We previously introduced the idea of our method [37]; it is further generalized and developed here, and some possible engineering applications are offered.

## 2. Proboscis

### 2.1. Structure

**Figure 1.**
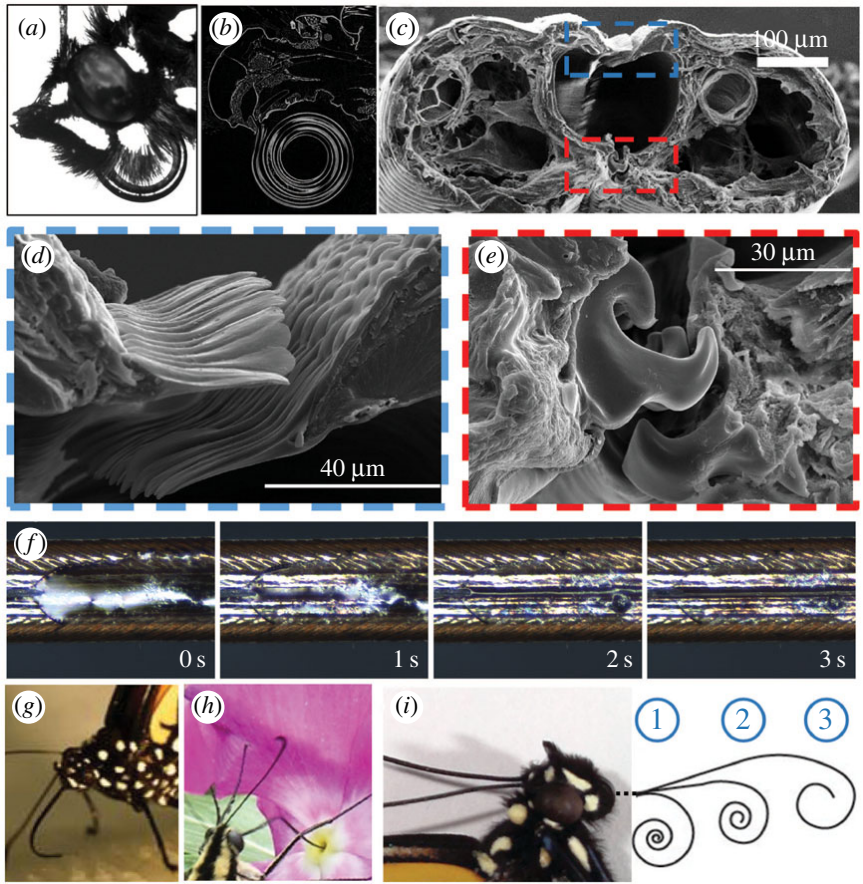
(*a*) Coiled proboscis of a monarch butterfly (*Danaus plexippus*). (*b*) Longitudinal section of the proboscis revealed by micro-CT. (*c*) Cross section of the proboscis of a hawk moth (*Manduca sexta*); areas in the dashed boxes are magnified in (*d*) and (*e*). (*d*) Dorsal legulae, showing their overlapping configuration. (*e*) Ventral legulae, showing their interdigitation. (*f*) Movement of methylene blue-dyed water through interlegular dorsal pores of the hawk moth (*Manduca sexta*) proboscis. (*g*) J-configuration of the proboscis of a monarch butterfly (*Danaus plexippus*). (*h*) Tiger swallowtail (*Papilio glaucus*) dipping its proboscis into a flower corolla. When the butterfly pulls the proboscis out, nectar remains on the external surface of the proboscis, which then moves to the permeable dorsal and ventral legular bands. (*i*) Monarch butterfly, showing schematic of different stages of proboscis coiling and uncoiling during feeding.

Micro-computed tomography (micro-CT) scans of the head and proboscis of the monarch butterfly (*Danaus plexippus*) were acquired using the technique described in [38]. The scan shows the complex three-dimensional structure of the butterfly feeding device (figure 1*b*). When the insect is not feeding, its proboscis is coiled in a spiral with multiple loops tightly wound one over the other (figure 1*a*), the number of loops varying from species to species.

The lepidopteran proboscis consists of two C-type fibres called galeae; when the insect emerges from the pupa, it assembles the galeae so that their C-shaped faces unite to form a food canal (figure 1*b,c*).

To acquire liquid and restrict entry of debris, Lepidoptera use a fence-like linking mechanism, called legulae, to hold the two galeae together and facilitate fluid entry (figure 1*d,e*). The legulae sit next to one another or overlap [10,11,17,18,39–41]. Experimental analysis of the wetting properties of the external surface of the proboscis reveals a hydrophobic–hydrophilic dichotomy [11]; about 5–20% of the proboscis length has a net hydrophilic surface, with the remaining 80–95% of each proboscis of the tested species having a net hydrophobic surface, except the two legular bands of the linking mechanism, which are hydrophilic [42].

### 2.2. Geometrical model of proboscis

**Figure 2.**
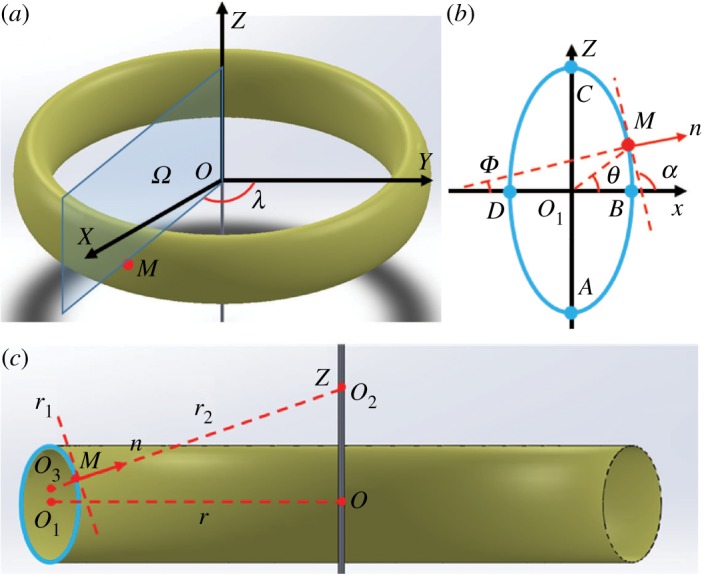
(*a*) A single loop of the proboscis is modelled as a ring of radius *r* with an elliptical cross section. Longitude *λ* is defined as the angle formed by the plane *Ω* and the plane *X* = 0. (*b*) Cross section of the ring formed by cutting it with plane *Ω*. (*c*) Definitions of principal radii of curvature *r*_1_ = *O*_3_*M* and $r_{2}=MO_{2}$of the ring surface at point *M*. The distance *OO*_1_ is equal to the ring radius *OO*_1_ = *r*. The proboscis cross section is assumed to be elliptical with semi-axes $O_{1}C=b$ and $O_{1}D=a$, where $O_{1}C⊥O_{1}O$ and $O_{1}D∥O_{1}O$.

We modelled a single loop of the proboscis as a ring of radius *r* (figure 2). Hereafter, the left cross section (figure 2*b,c*) is used as the reference; hence, all angles and positions will be evaluated according to the left cross section. When r→+∞, the model describes a straight proboscis. The notations are presented in table 1.

**Table 1.** Notations.

*M* (*x*, Z)	point on the ring surface defined in figure 2*a*
*Ω*	plane passing through point *M* and the *OZ* axis, defined in figure 2*a*
*λ*	the longitude angle formed by plane *Ω* and plane *X* = 0, defined in figure 2*a*
*ϕ*	the latitude angle formed by vector ***n*** and plane *Z* = 0, defined in figure 2*b*
*O*	centre of the ring, defined in figure 2
*r*_1_, *r*_2_	two principal radii of curvature at point *M* defined in figure 2*c*
*O*_2_, *O*_3_	centres of two radii *r*_1_, *r*_2_, respectively, defined in figure 2*c*
*O*_1_	centre of ring cross section, defined in figure 2*a*
*r*	ring radius defined in figure 2*c*
***n***	outward unit normal vector at point *M*, defined in figure 2*c*
*a*, *b*	two semi-axes of an ellipse in figure 2*b*
*φ*	the auxiliary angle used for parametrization of the local coordinates (*x*, *Z*)
*ρ*	radius in polar system of coordinates (*ρ*, *θ*)
*θ*	polar angle defined in polar system of coordinates (*ρ*, *θ*)
*α*	angle formed by the tangent line passing through point *M* and the *x*-axis (definition in figure 2*b*)
*e* = *b*/*a*	ellipticity of cross section
*h*	thickness of liquid film deposited on ring surface
*P*_l_	pressure inside a liquid film
*P*_a_	atmospheric pressure
*R* = *r*/*a*	normalized *r*
*R*_1_ = *r*_1_/*a*	normalized *r*_1_
*R*_2_ = *r*_2_/*a*	normalized *r*_2_
$P=(P_{l}-P_{a})\;a/\sigma$	dimensionless pressure difference
$\Delta P=(P_{l}^{\mathrm{local}\;\min}-P_{l}^{\mathrm{absolute}\;\min})\;a\text{/}\sigma$	normalized pressure difference between local and absolute minima
*θ*_c_	critical polar angle defining the position of attractors in figure 10
*e*_c_	critical cross section ellipticity to reach the maximum *θ*_c_

The position of any point *M* sitting on the ring surface is conveniently specified by a Cartesian system of coordinates (*X*, *Y*, *Z*), with the *Z*-axis taken perpendicular to the ring plane (*X*, *Y*). The centre of this (*X*, *Y*, *Z*) coordinate system is placed in the ring centre *O*. The ring surface is created by rotating an ellipse with semi-axes *a* and *b* around the *Z*-axis. It is convenient to introduce a local system of coordinates (*x*, *Z*) (figure 2*b*), with its centre placed at the centre of the ellipse *O*_1_. Thus, the profile of this ellipse is defined by the formula $(x^{2}\text{/}a^{2})+(Z^{2}\text{/}b^{2})=1$.

One can introduce an alternative system of coordinates specifying the position of point *M* on the ring surface by latitude *ϕ* and longitude *λ*. The latitude *ϕ* is measured by the angle formed by the outward normal vector ***n*** at point *M* and the ring plane *Z* = 0. We introduce the plane *Ω* containing the vector ***n*** at point *M* and axis *OZ.* The longitude *λ* is defined as the angle formed by the plane *Ω* and reference plane, *X* = 0.

All points on the proboscis surface with the same latitude *ϕ* form a closed curve called the parallel. All points on the proboscis surface with the same longitude *λ* form a closed curve called the meridian. Meridians and parallels constitute a parametric grid on the proboscis surface, with the ellipses as meridians and circles as parallels. Point *M*, for example, belongs to the meridian formed by cutting the ring by the plane *Ω* (figure 2*a,b*). Using the latitude and longitude, we can make an alternative parametrization specifying the position of point *M* as (*ϕ*, *λ*).

There is another convenient parametrization with an auxiliary angle 0<φ<2π such that x=a⁡cos⁡φ, Z=b⁡sin⁡φ. With this parametrization, the local coordinates of point *M*(*x*, *Z*) on this ellipse are expressed as *M*(a⁡cos⁡φ, b⁡sin⁡φ). Alternatively, this point can be specified in the local polar system of coordinates *M*(*ρ*(*θ*),*θ*), with angle *θ* formed by the line *MO*_1_ with the ring plane *Z* = 0; the radius *ρ*(*θ*) is defined from equation $\rho (\theta )=ab{\left({\left(b\text{cos}\theta \right)}^{2}+{\left(a\text{sin}\theta \right)}^{2}\right)}^{-1/2}$. In the polar system of coordinates, the vertexes of the ellipse are identified by angle *θ*: at point *A*, θ=−π/2, at point *B*, *θ* = 0, at point *C*, *θ* = *π*/2 and at point *D*, *θ* = *π* (figure 2*b*).

The tangent plane at point *M* is associated with the normal vector ***n*** at point *M* (figure 2*b,c*). The tangent plane is perpendicular to the normal vector ***n*** and makes angle *α* with the ring plane *Z* = 0, figure 2*b*. Thus, the angle *α* defining the slope of the ellipse at point *M*, tan *α*, can be found through derivative tan⁡α=dZ/dx, and can be further expressed through angle *φ* by applying the chain rule of differentiation with x=acos⁡φ, Z=bsin⁡φ as:

2.1 $$\text{tan}\alpha =\frac{\text{d}Z}{\text{d}x}=\frac{\text{d}Z}{\text{d}\varphi }\cdot \frac{\text{d}\varphi }{\text{d}x}=-\frac{b}{a}\cot\varphi =-e\cot\varphi .$$

The angle *θ* of the polar system of coordinates and the auxiliary angle *φ* are connected through the following relationship:

2.2 $$\text{tan}\;\theta =\frac{Z}{x}=\frac{b\sin\varphi }{a\cos\varphi }=e\text{tan}\varphi .$$

In equations (2.1) and (2.2), the proboscis ellipticity *e* is introduced as *e* = *b*/*a*. All proboscises elongated along the *Z*-axis have ellipticity greater than 1, *e* > 1, and all proboscises elongated in the ring plane *Z* = 0 have *e* < 1.

## 3. Laplace law of capillarity applied to a thin coating film

Each time the insect dips its proboscis into a flower, it picks up a thin, external layer of nectar. The surface of the liquid film is subject to the action of surface tension *σ*. The thickness of the liquid film *h* is small, so that it is safe to assume that gravity plays an insignificant role and only capillary force is important [11,37]. Field observations suggest that the thickness *h* of the nectar layer is much smaller than all characteristic dimensions of the proboscis, h≪a, h≪b, h≪r. These values vary with different proboscises, but typically *h* is at the level of 10^−6^ m, *a* and *b* are at the level of 10^−4^ m and *r* is at the level of 10^−3^ m [1]. Therefore, film non-uniformity can be neglected, assuming that the air–liquid interface *h*(*X*, *Y*, *Z*) is positioned equidistant to the proboscis surface; in other words, at any longitude *λ*, the film profile is specified by equation $(x^{2}\text{/( }a+h{\text{) }}^{2})+(Z^{2}\text{/}{\left(b+h\right)}^{2})=1$. Because the film is thin, h≪a, h≪b, one can estimate the pressure distribution over the air–liquid interface by applying the Laplace Law of capillarity directly to the proboscis surface, $(x^{2}\text{/( }a+h{\text{) }}^{2})+(Z^{2}\text{/}{\left(b+h\right)}^{2})\approx (x^{2}\text{/}a^{2})+(Z^{2}\text{/}b^{2})\approx 1$. The Laplace law of capillarity states that the pressure under the film surface *P*_l_ differs from atmospheric pressure *P*_a_, and the pressure differential is determined by the surface tension *σ* and the mean curvature of the surface as

3.1 $$P_{l}-P_{a}=\sigma \left(\frac{1}{r_{1}}+\frac{1}{r_{2}}\right),$$

where *r*_1_ and *r*_2_ are the principal radii of curvature of the liquid–air interface, which, in a first approximation, coincides with the proboscis surface.

We distinguish the behaviour of *internal films* coating the walls of the food canal from that of *external films* coating the external surface of the proboscis. To understand the differences in pressure distributions for external and internal films, one has to bear in mind that the sign of curvature in the Laplace law is associated with the outward normal vector to the given point at the surface [43]. Because the directions of outward normal vectors for the internal and external films are different, the capillary pressures also differ.

According to differential geometry [43], the circle centres *O*_2_ and *O*_3_, defining the principal radii of curvature, are sitting on the line obtained by continuation of the outward normal vector ***n*** to the surface at point *M*. For any axisymmetric surface, one centre of these circles must be sitting on the *Z*-axis. In figure 2*c*, this centre of curvature is *O*_2_. Another centre can be located anywhere along the line *MO*_2_. In figure 2*c*, the two principal radii of curvature for the ring surface are denoted as $MO_{3}=r_{1}$ and $MO_{2}=r_{2}$, respectively. We define the radii of curvature *r*_1_ and *r*_2_ as positive when the vectors ***MO***_2_, ***MO***_3_ are pointing towards the *Z*-axis.

For convenience of calculation and analysis, we normalize all geometric parameters by the distance *a*. Therefore, the dimensionless radius of the ring is

3.2 $$R=\frac{r}{a}$$

and the principal radius of curvature of the meridians is $R_{1}=MO_{3}\text{/}a=r_{1}\text{/}a$. The radius *R*_1_ is calculated by using the definition of curvature of a plane curve from differential geometry [43]:

$$R_{1}=\frac{r_{1}}{a}=a^{-1}\left(\frac{{\left(1+{\left(\text{d}Z/\text{d}x\right)}^{2}\right)}^{3/2}}{\left|{\text{d}}^{2}Z/\text{d}x^{2}\right|}\right)=\frac{{\left(a^{4}-a^{2}x^{2}+b^{2}x^{2}\right)}^{3/2}}{a^{5}b}.$$

Inputting x=a⁡cos⁡φ, Z=b⁡sin⁡φ, *e* = *b*/*a*, we finally obtain

3.3 $$R_{1}=\frac{r_{1}}{a}=\frac{{\left(\sin^{2}\varphi +e^{2}\cos^{2}\varphi \right)}^{3/2}}{e}.$$

When an observer moves along the meridian, the radius of curvature changes from one position to the other.

The second principal radius of curvature $r_{2}=MO_{2}$ is calculated using the geometrical construction in figure 2*c*. For the two intervals θ∈[−π/2, π/2] and θ∈[π/2, 3π/2], the expressions of *r*_2_ are different:

3.4 $$R_{2}=\frac{r_{2}}{a}=\frac{\left(r-|a\cos\varphi |\right)}{a\sin\alpha }=\frac{\left(R-|\cos\varphi |\right)}{\sin\alpha },\;\theta \in \left[-\frac{\pi }{2},\frac{\pi }{2}\right]$$

and

3.5 $$R_{2}=\frac{r_{2}}{a}=\frac{\left(r+|a\cos\varphi |\right)}{a\sin(\alpha -\pi )}=-\frac{\left(R+|\cos\varphi |\right)}{\sin\alpha },\;\theta \in \left[\frac{\pi }{2},\frac{3\pi }{2}\right].$$

Substituting these equations into the Laplace law of capillarity, equation (3.1), we obtain two different representations of the dimensionless pressure difference, $P=a(P_{l}-P_{a})\text{/}\sigma$, for a film coating the food canal and for a film coating the external surface of the proboscis.

Film coating the external surface of the elliptical proboscis:

3.6 $$P=\frac{(P_{l}-P_{a})a}{\sigma }=\frac{e}{{\left(\sin^{2}\varphi +e^{2}\cos^{2}\varphi \right)}^{3/2}}-\frac{\sin\alpha }{\left(R-\cos\varphi \right)}.$$

Film coating the internal surface of the elliptical food canal:

3.7 $$P=\frac{(P_{l}-P_{a})a}{\sigma }=-\left(\frac{e}{{\left(\sin^{2}\varphi +e^{2}\cos^{2}\varphi \right)}^{3/2}}-\frac{\sin\alpha }{\left(R-\cos\varphi \right)}\right).$$

These two formulae together with equations (2.1) and (2.2) describe the pressure distribution over the liquid–air interface of a film covering either the external surface of the proboscis or the wall of the food canal. Two limiting cases of a straight proboscis and a proboscis loop made of a circular cylinder provide the physical background to illustrate a counterintuitive behaviour of liquid films on elliptical proboscises.

## 4. Behaviour of liquid films on straight elliptical proboscises

As the ring radius goes to infinity, R→+∞, equations (3.6) and (3.7) are simplified to

4.1 $$P=\frac{a(P_{l}-P_{a})}{\sigma }=\frac{e}{{\left(\sin^{2}\varphi +e^{2}\cos^{2}\varphi \right)}^{3/2}}$$

and

**Figure 3.**
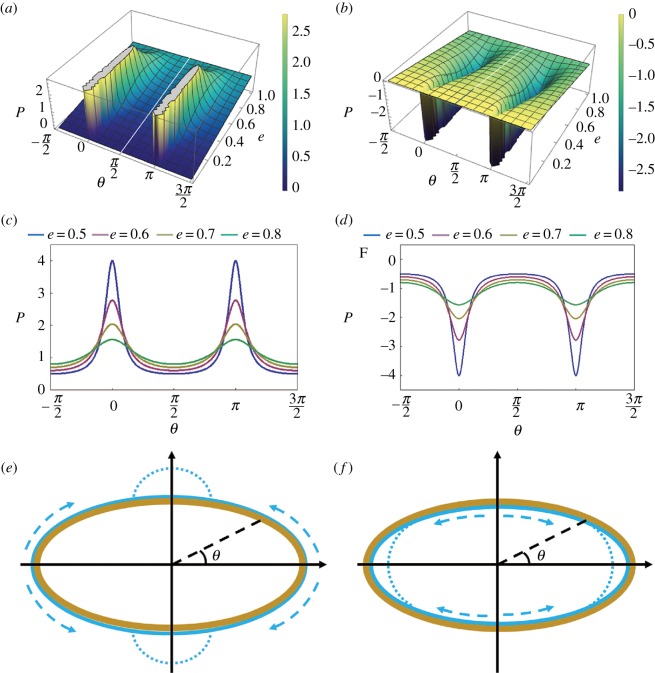
(*a*) Pressure distribution in the external film on a straight elliptical proboscis. (*b*) Pressure distribution in the internal film on a straight elliptical food canal. (*c*,*d*) Schematics showing positions where liquid is expected to flow and collect. (*c*) The external film is expected to collapse into two semi-cylindrical columns at the flattened sides of the proboscis. (*d*) The internal film is expected to flow towards the most curved sides of the food canal where it will form two liquid menisci. (*e*,*f*) Schematics of surface activity showing fluid flow and collection, (*e*) externally at flatter regions and (*f*) internally at more curved regions.

4.2 $$P=\frac{a(P_{l}-P_{a})}{\sigma }=-\frac{e}{{\left(\sin^{2}\varphi +e^{2}\cos^{2}\varphi \right)}^{3/2}},$$

respectively. These pressure distributions are plotted in figure 3*a,b* for external and internal films, respectively. Figure 3*e,f* are the schematics showing where the liquid should be collected based on the pressure distribution predictions in figure 3*c,d*, respectively.

This model of films is important to understand scenarios of food flow in the straightened proboscises of Lepidoptera. In contrast to the circular proboscis where the pressure in the film is uniform along the circumference, this model emphasizes the effect of non-uniformity or circumferential distribution of capillary pressure that is spontaneously built on an elliptical surface of the proboscis. The model suggests that as soon as a liquid film is deposited on the proboscis surface, the fluid is prone to move towards the dorsal and ventral legular bands where it can be collected and then enter the food canal.

**Figure 4.**
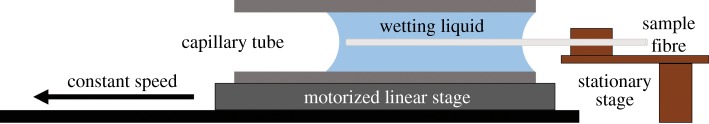
Experimental set-up used in proboscis- and wire-coating experiments. The tube with the wetting liquid is moved at a constant speed, 1 mm s^−1^, and the meniscus leaves behind a film coating the fibre surface.

To examine our hypothesis that liquid can be passively collected at the permeable dorsal and ventral legular bands due to proboscis ellipticity, an experiment (figure 4) was designed to observe film development after coating a fibre. We experimented with straight proboscises of *Manduca sexta*. In additional experiments, we used a solid SUS-304 stainless steel wire of elliptical cross section (*a* = 300 µm, *b* = 200 µm) without any holes to suck in the liquid. The presence of the film and its thickness were evaluated using a dry elliptical wire (figure 6*a*) as a reference.

**Figure 5.**
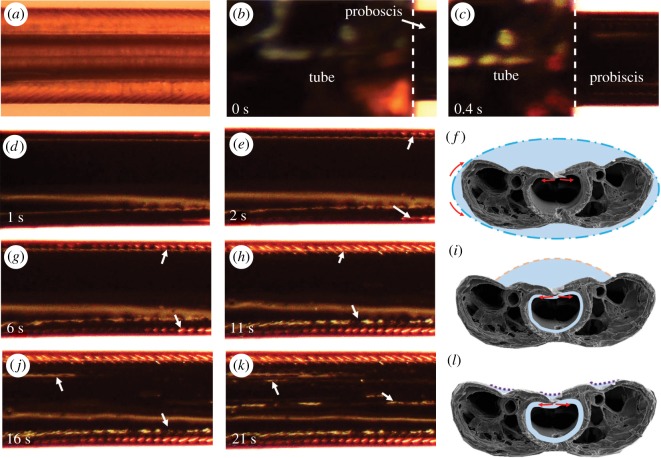
Dewetting of the proboscis of *Manduca sexta* by an ink film. (*a*) A dry, straightened proboscis before it is coated with ink. The dorsal legular band running along the proboscis appears darker compared with the rest of the proboscis. (*b*,*c*) Film deposition process. The tube of ink (left of the dashed line) is moved to the left and a black film is deposited on the proboscis. (*d*,*e*) After the film forms, it flows from the sides to the centre (i.e. towards the legular band). (*d*) The proboscis is completely covered with the film. (*e*) Two contact lines (shown by arrows) form after dewetting of the proboscis sides. (*f*) Schematic of the movement of the external film in images (*d*) and (*e*). (*g*,*h*) The contact lines recede from the proboscis sides towards the legular band. (*i*) Schematic of the movement of liquid in images (*g*) and (*h*). ( *j*,*k*) The legular bands dewet as the film moves into the food canal. The bright bands (shown by arrows) are the grooved features of this band shown in detail in figure 1*c*. (*l*) Schematic of the movement of liquid in images ( *j*) and (*k*).

The proboscis of *Manduca sexta* was straightened, dried at room temperature for 48 h (figure 5*a*), and inserted into a capillary tube (inner diameter of 1.6 mm), filled with black ink (Radiant™ Colors Turbo Black). It was then exposed by moving the tube at 1 mm s^−1^ with linear positioning stages (VT-21, Micos) to the left while filming from the top using a high-speed camera (POINTGREY® FL3-U3-13S2C-CS) and microscopic lens (Meiji Techno® Short UNIMAC MacroZoom Lense MS-40); thus, we were able to magnify the proboscis and distinguish the flow features (figure 5). Once the proboscis leaves the tube, its surface is covered with a film of ink (figure 5*b,c*). The film gradually flows from the sides with a smaller radius of curvature towards the dorsal legular band, leaving the proboscis sides dewetted (figure 5*d,e,g,h*). The arrows in these figures mark the two contact lines receding towards the dorsal legular band. At the same time, liquid is drawn into the food canal through the porous legular band; the arrows in figure 5*j,k* show the two boundaries of the legular band that become visible through the ink.

**Figure 6.**
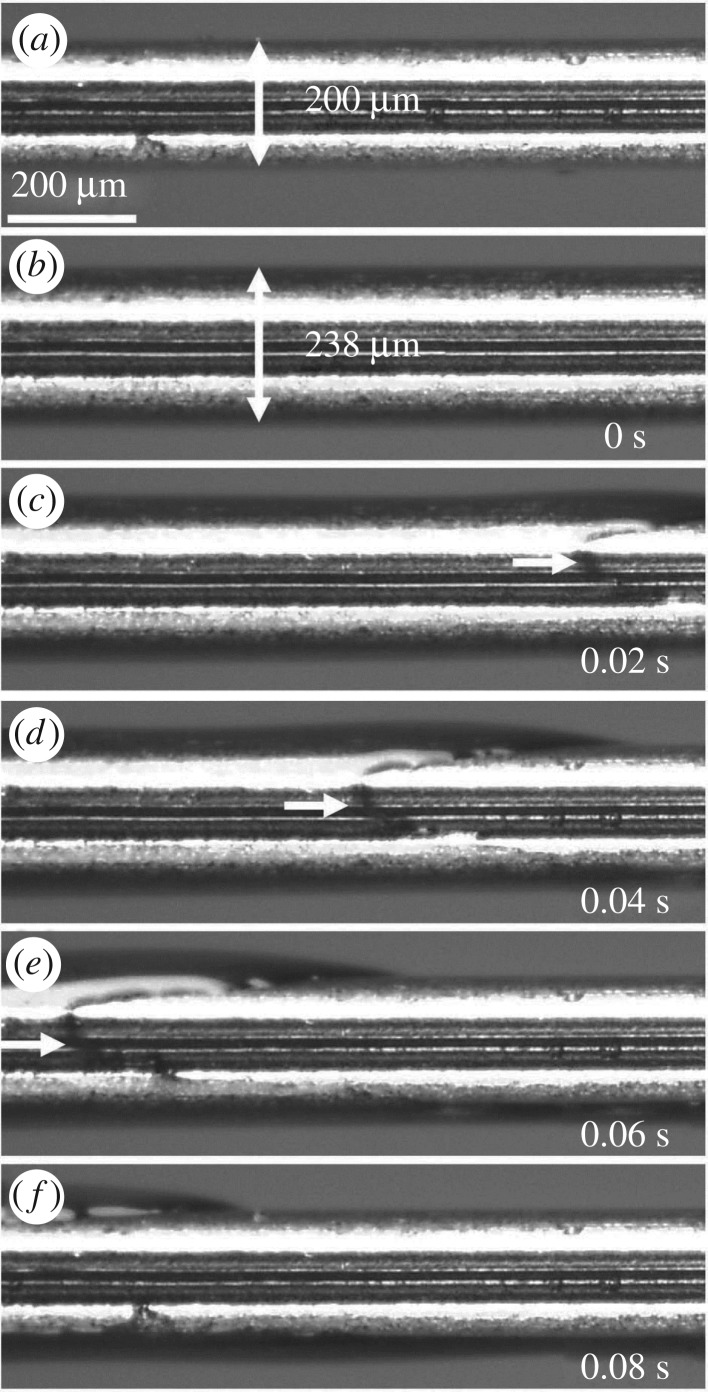
Instability of a glycerin film deposited on an elliptical wire. The narrow side is facing the camera. (*a*) Dry wire before film deposition. (*b*) Wire with a film after its deposition. (*c*) Formation of a contact line (shown by arrow). (*d–f*) The main features of the process of film break-up and separation into two clam-shell droplets sitting on the wider parts of the wire.

To reveal the contribution of dorsal legulae to the development of film instability, we conducted the same experiment with an elliptical wire. The film of ink broke up too quickly on the wire to document the process with available cameras. We, therefore, applied glycerin, which is about 1000 times thicker than the ink. Yet, the wetting properties of the glycerin–wire pair are similar to those of the ink–proboscis pair (figure 6). Glycerin deposition on the proboscis showed the same features as those with the ink in figure 5. The film was broken, with formation of four almost straight contact lines receding towards the two legular bands. However, visualization of the dewetting processes with glycerin is challenging because of weak optical contrast. On the other hand, the wire surface is shiny and allows visualization of flow of the glycerin film. Our experiments deal with static properties of liquid films; thus, viscosity does not affect the results. In addition, pressure distribution is linearly proportional to surface tension; therefore, the ratio of pressure to surface tension that we examined should not be affected by surface tension.

The wire was covered with a film of glycerin after withdrawal from a capillary tube (figure 6*b*). Within 0.1 s, the film lost its stability and broke into islands, with the liquid at the narrow side flowing to the wide sides of the wire, as shown by the observed contact line (white arrow in figure 6*c–e*). The film finally broke into two parts sitting on each of the wide sides, forming clam-shell droplets (figure 6*f*).

The flow patterns for the proboscis and the wire are similar but bear distinguishable features. In both experiments, as theory predicts, liquid from the most curved parts of the fibres moved to the less curved sides. However, the film of the less viscous ink was more stable on the proboscis and moved along meridians over the entire visible length of the proboscis, forming a long liquid column (figure 5). Finally, the film moved completely into the food canal through the legular band. In contrast to figure 1*f*, when a droplet was applied directly onto the legular band and was pinned at the contact line, the coating film initially enveloped the entire proboscis. The final stage was the same: complete absorption of the droplet and film by the food canal.

The film deposited on elliptical wires could not move along meridians over the entire length of the wire. Instead, some liquid bands at the most curved parts flowed faster. In figure 6, these bands are located at the right ends of the viewing regions. Once some islands on the wire surface were dewetted a meniscus formed, which moved to the left, uncovering the rest of the surface. Finally, the liquid from the most curved parts was fully drawn away and formed a series of clam-shell droplets (figure 6) at the wide sides; the liquid bridges connecting them disappeared (figure 6*f*). This scenario can explain the appearance of droplets on proboscises of long-tongued butterflies and moths (e.g. [44, figs 8 and 10]), where nectar droplets on the proboscis of a hovering moth are visible. The observed pattern of break-up of liquid films on elliptical fibres is probably common for many natural fibres including ribbon-like fibres such as grass leaves and short lepidopteran proboscises. For these fibre shapes, the clam-shell droplets are formed when the contact angle is greater than 38.24°, i.e. when the droplet is supposed to bead up on the ribbon surface [45]. The clam-shell droplets are easily shaken off, demonstrating that the natural design of elliptical fibres helps maintain surface cleanliness.

## 5. Behaviour of liquid films on coiled circular proboscises

Consider a single loop of a circular proboscis, *e* = 1. In contrast to the straight proboscis of circular cross section, for which the pressure in the film is uniform along the circumference, the pressure becomes circumferentially non-uniform when the proboscis is coiled. Taking into account equations (3.6) and (3.7), we obtain the following for the limit as *e* = 1.

Film coating the external surface of the elliptical proboscis:

5.1 $$P=\frac{(P_{l}-P_{a})a}{\sigma }=1-\frac{\sin\alpha }{\left(R-\cos\varphi \right)}.$$

Film coating the internal surface of the elliptical food canal:

5.2 $$P=\frac{(P_{l}-P_{a})a}{\sigma }=-\left(1-\frac{\sin\alpha }{\left(R-\cos\varphi \right)}\right).$$

**Figure 7.**
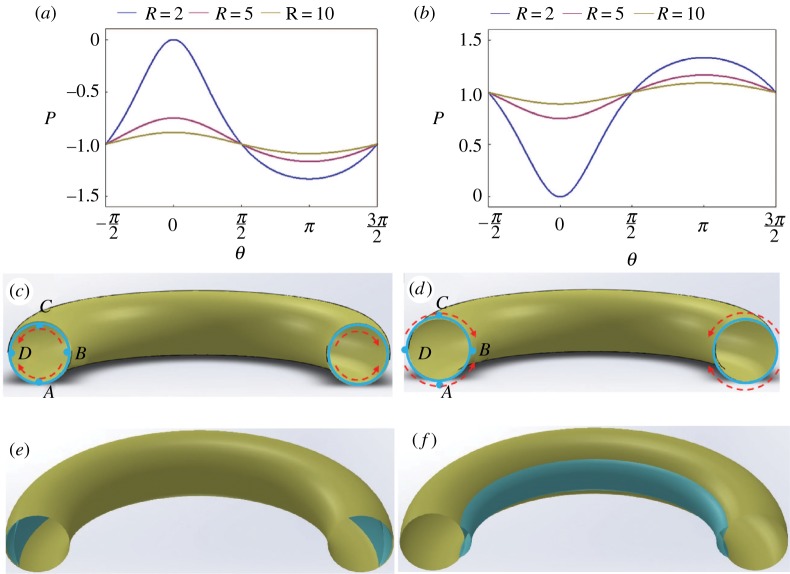
(*a*) Pressure distribution in the film coating the food canal; pressures for three different loop radii are shown. (*b*) Pressure distributions in the films coating the proboscis exterior for three different loop radii. (*c*,*d*) Schematics showing the flow directions for the internal (*c*) and external (*d*) films. (*e*,*f*) Schematics showing the possible final configurations of the internal (*e*) and external (*f*) films. In theory, they should be shaped as unduloids [46].

Comparison of equations (5.1) and (5.2) and equation (3.3) reveals that the contribution of surface tension acting along the meridians to the capillary pressure is constant along the proboscis; for the external film, this contribution to the pressure difference is written in dimensional form as ${\left(P_{l}-P_{a}\right)}_{\mathrm{meridian}}=\sigma \text{/}a$, and for the internal film it is ${\left(P_{l}-P_{a}\right)}_{\mathrm{meridian}}=-\sigma \text{/}a$. This contribution is equal to the capillary pressure in a straight liquid cylinder (where pressure in the liquid is greater than the reference atmospheric pressure) or outside a cylindrical bubble (where pressure in the liquid is smaller than the reference atmospheric pressure in the bubble). Thus, the non-uniformity of the capillary pressure in equations (5.1) and (5.2) comes solely from the bending of the proboscis. In figure 7*a*, we plot the distribution of capillary pressure that is spontaneously generated in the internal film when the insect coils its proboscis. Figure 7*b* illustrates the effect of proboscis coiling on the external film, provided that in both cases the film coats the respective surfaces of the proboscis uniformly.

According to figure 7*a,b*, the capillary pressure is minimal at the parallel *θ* = *π* for the internal film (point *D* in figure 7*c*) and at the parallel *θ* = 0 for the external film (point *B* in figure 7*d*). These two parallels are identified as liquid attractors. Hence, the liquid will tend to flow spontaneously towards these attractor parallels as the most favourable locations for it to be collected, as shown in figure 7*e,f*. These considerations help to understand the effect of concave–convex bends on the pressure distribution in the film.

**Figure 8.**
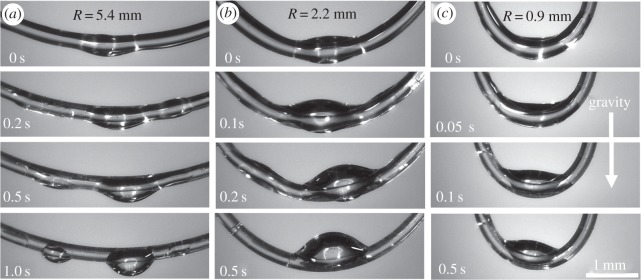
Evolution of a thin glycerin film deposited on a circular loop with different radii of curvatures. (*a*) 5.4 mm, (*b*) 2.2 mm and (*c*) 0.9 mm. The film develops into a droplet collecting the film fluid on different regions of the loop.

As an illustration of these findings, we traced the evolution of thin glycerin films deposited on nylon fishing line (Berkley Trilene® Super Strong™, diameter 0.28 mm) looped for different radii of curvature (figure 8). Using the capillary rise experiment as discussed in [32], we obtained the contact angle of 31° for glycerin on these fibres. The loops were vertically withdrawn from a glycerin reservoir with the liquid completely covering the entire surface. The Bond numbers of drops formed, $Bo=\rho gV^{2/3}/\sigma$, where *ρ* is liquid density, *g* is acceleration due to gravity, and *V* is droplet volume, serve as the measures of importance of droplet weight with respect to the capillary forces. Estimating the volume of the drop from figure 8*a*, we obtained its volume as approx. 2 µl. Inputting *ρ *= 1.26 × 10^3^ kg m^−3^, *σ *= 64 × 10^−3^ N m^−1^, the resulting Bond number is estimated as *Bo* = 0.3. Thus, the effects of droplet weight and surface tension are comparable to each other, so that they both play important roles in the observed phenomenon shown in figure 8. When the radius of curvature of the loop is large (figure 8*a*), the capillary pressure differential is small (figure 7), so that the effect of gravity dominates and the drops roll over to the bottom of the loop. In contrast to this case, when the radius of the loop decreases, the induced capillary pressure differential increases pushing the film to move towards the loop interior, as theory predicts. This capillary force is so strong that gravity can be defeated and the droplet forms on the internal side of the loop (figure 8*b,c*).

## 6. Pressure distribution in external liquid films on a coiled elliptical proboscis

The two analysed limiting cases, a straight elliptical and a coiled circular proboscis, establish the groundplan for analysis of insect feeding behaviour. The Laplace law of capillarity imposes a constraint on the uniformity of circumferential distribution of pressure in elliptical proboscises, as well as in coiled circular proboscises. Thus, the direction of cross-sectional elongation of the proboscis in the coiled state affects the attractor positions. Below, we evaluate the effect of coiling on liquid distribution for elliptical proboscises. Two cases of orientation of proboscis cross section, with the ellipse elongation perpendicular to the coil plane (i.e. ellipticity *e* > 1), and with the ellipse elongation parallel to the coil plane (*e* < 1) are discussed separately. In both cases, the food canal is considered circular (*e* = 1). Therefore, the results of §5 hold for the internal film coating the walls of the food canal.

We apply equations (2.1), (2.2) and (3.6) for the pressure distribution analysis.

### 6.1. Case *e* > 1

**Figure 9.**
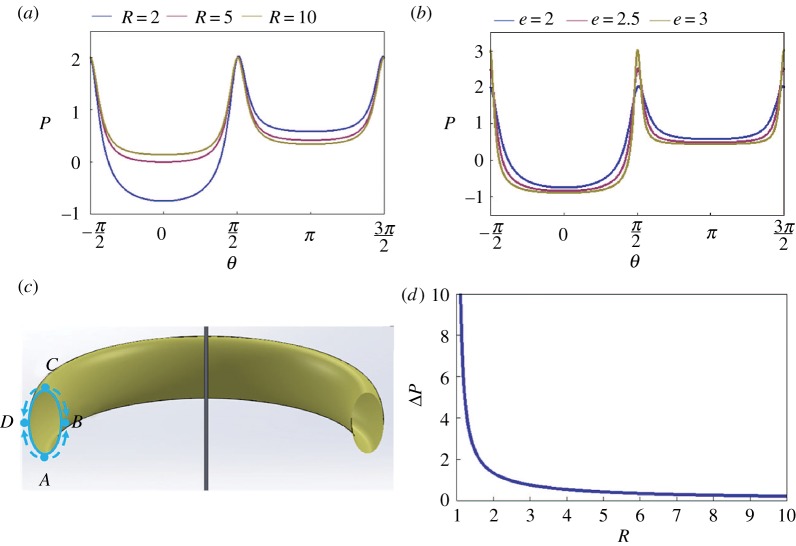
(*a*) Dimensionless pressure difference *P* versus angle *θ* for different dimensionless coil radii *R* at fixed ellipticity *e* = 2. (*b*) Dimensionless pressure difference *P* versus angle *θ* for different ellipticities at the fixed dimensionless coil radius *R* = 2. (*c*) Schematic of liquid transport from the proboscis sides, parallels *A* and *C*, towards the ventral and dorsal legular bands, parallels *B* and *D*, respectively. (*d*) Dimensionless pressure differential of the absolute minimum and local minimum, $\Delta P=(P_{l}^{\mathrm{local}\;\min}-P_{l}^{\mathrm{absolute}\;\min})a\text{/}\sigma$, as a function of the dimensionless coil radius of the proboscis model.

The effect of ring radius *R* and cross-section ellipticity *e* on the pressure distribution in the film is illustrated in figure 9*a,b*. The dimensionless pressure difference *P* is plotted as a function of the polar angle *θ*. Compared to the film on a circular proboscis where the pressure has only one minimum and one maximum (figure 7), the film on an elliptical proboscis has a more complex pressure distribution. There are two local minimums and two local maximums. The ventral and dorsal legular bands are the attractors for the liquid; that is, the pressure at these parallels has a local minimum. The ventral legular band *B*, *θ* = 0 (figure 9*c*) is always more attractive, as it has the lowest pressure. The parallels *A* and *C*, θ=±π/2, have maximum pressure and, hence, liquid moves from these parallels towards the ventral or dorsal legular bands.

Although the location of the attractor parallels is not influenced by the value of parameters *R* and *e* (figure 9*a,b*), the driving pressure differential might be affected by these parameters. We, therefore, examined the dependence of the driving pressure differential $\Delta P=(P_{l}^{\mathrm{local}\;\min}-P_{l}^{\mathrm{absolute}\;\min})a\text{/}\sigma$ on these parameters:

6.1 $$\Delta P=\frac{(P_{l}^{\mathrm{local}\;\min}-P_{l}^{\mathrm{absolute}\;\min})a}{\sigma }=\frac{1}{\left(R+1\right)}+\frac{1}{\left(R-1\right)}.$$

This pressure differential does not depend on proboscis ellipticity. Figure 9*d* summarizes the results of calculations of Δ*P* for different coil radii.

### 6.2. General case 0<e<∞

**Figure 10.**
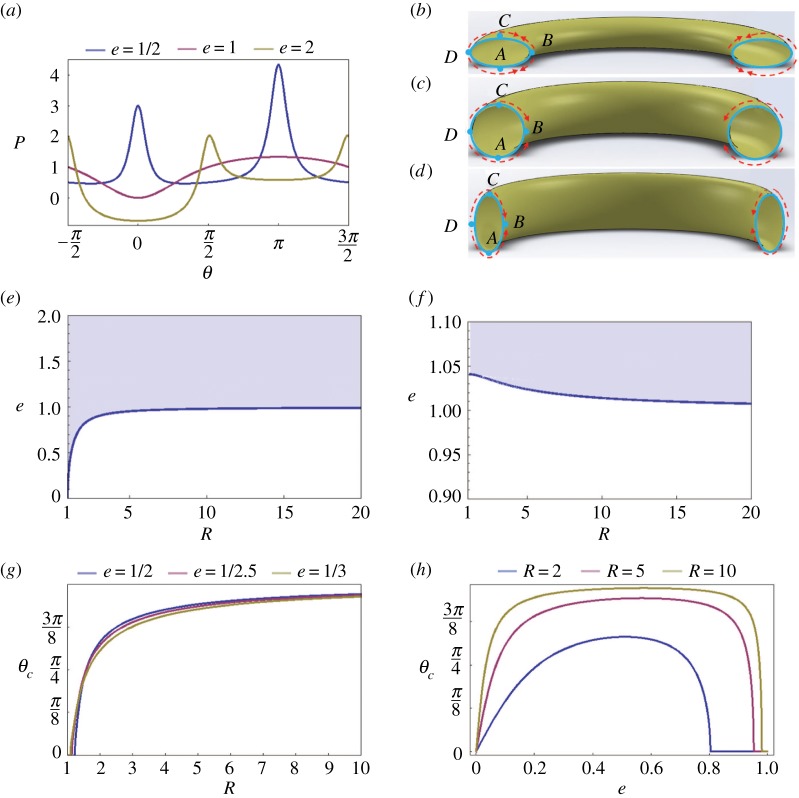
(*a*) Pressure distribution in films deposited on the external wall of a proboscis with *e* = 1/2, 1 and 2 coiled in a ring of radius *R* = 2. (*b–d*) Schematic showing flow inside the external liquid film on three types of proboscises with *e* = 1/2, 1 and 2, illustrating the results of (*a*). (*e*) Diagram of transition from a single attractor at *θ* = 0 (shaded region) to two attractors (white region). (*f*) Diagram of transition from a single attractor at *θ* = *π* (shaded region) to two attractors (white region). (*g*) Location of one of the mirror-symmetrical attractors specified by polar angle *θ*_c_ as a function of the dimensionless ring radius *R* for the three different proboscis ellipticities. (*h*) Location of one of the mirror-symmetrical attractors specified by polar angle *θ*_c_ as a function of proboscis ellipticity for the three different ring radii *R*.

In figure 10*a*, we plot the pressure distribution over the liquid film deposited on the external walls of proboscises with different ellipticity *e*. The pressure profile changes drastically when the orientation of proboscis elongation changes from the case discussed in §6.1 to the case when proboscis elongation flips to become parallel to the plane of proboscis coiling, as in figure 10*b*, with the ellipticity less than one, *e* < 1. As an illustration, in figure 10*a*, we plot the pressure profile for the proboscis with ellipticity *e* = 1/2. The graph suggests that the pressure reaches the two local minimums at the mirror-symmetrical positions shifted from parallel *B* towards parallels *A* and *C* (figure 10*b*). Thus, parallel *B* is no longer a single attractor for the liquid; the liquid collection can occur at the two mirror-symmetrical parallels.

The transition from a single attractor to two attractors with the same absolute minimum pressure occurs when the pressure at the parallel *θ* = 0 flips from a minimum to a maximum. Mathematically, this transition happens when the pressure profile passes an inflection point; that is, the second derivative of equation (3.6) with respect to *θ* turns to zero. The same arguments are applied to the pressure at the parallel *θ* = *π*. These derivatives give us two quadratic equations

6.2 $$\frac{3(e^{2}-1)}{e^{4}}{\left(R-1\right)}^{2}+\frac{1}{e^{2}}(R-1)+1=0,\;\text{criterion for transition at }\;\theta =0$$

and

6.3 $$\frac{3(e^{2}-1)}{e^{4}}{\left(R+1\right)}^{2}+\frac{1}{e^{2}}(R+1)+1=0,\;\text{criterion for transition at }\;\theta =\pi .$$

By solving these two equations, we can plot the diagrams specifying a transition from a single attractor to the two attractors that identify the potential location of the liquid columns (figure 10*e,f*).

These two parallels–attractors are specified by two angles $\pm \theta_{c}$. Their location is influenced by the ring radius *R* and the proboscis ellipticity *e* as shown in figure 10*g,h*. At the given ellipticity *e*, the attractor position *θ*_c_ increases along with the coil radius *R* (figure 10*g*), approaching θ=π/2 as the radius *R* goes to infinity and the proboscis straightens out.

At the given coil radius *R*, the attractor position *θ*_c_ changes non-monotonically with *e*, having a well-pronounced maximum (figure 10*h*). This non-trivial result deserves special analysis.

When *e* is close to 1 (i.e. the proboscis is close to circular), the attractors $\pm \theta_{c}$ should be positioned close to the parallel *B*, $\theta_{c}\to 0$ . This explains why the attractor angular location goes to zero, $\theta_{c}\to 0$ (ribbon-like, figure 10*d*), when the ellipticity is finite. Once the attractor hits $\theta_{c}=0,$ further increase of proboscis ellipticity *e* does not change the attractor position (figure 10*e*).

**Figure 11.**
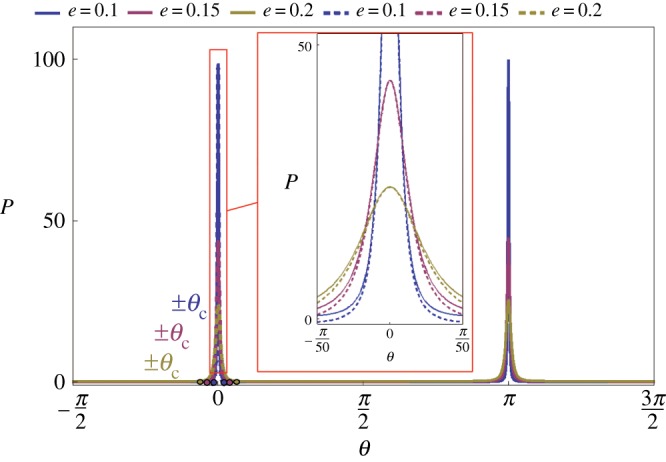
Pressure distribution in a film coating a washer-like proboscis with loop radius *R* = 2. The pressure is almost zero at the entire surface of this type of proboscis and it spikes up at the edges. The dashed lines correspond to the asymptotic solution, equation (6.4). The small circles on the horizontal axis near *θ* = 0 show the attractor position for each proboscis.

We must turn to the opposite limit as *e* goes to zero. In this case, the proboscis coil looks like a thin washer with nearly flat sides and sharp edges (figure 10*b,e*). On the washer sides, the capillary pressure goes to zero because the film is almost flat (figure 11). Therefore, we need to study pressure behaviour near the edges *B* and *D*.

The behaviour of pressure in the vicinity of parallel *B* can be studied asymptotically using equation (3.6). In the vicinity of parallel *B*, one can asymptotically evaluate the *α* and *φ* parameters from equations (2.1) and (2.2) as α≈π/2, φ≈θ/e, provided that θ→0. Substituting these parameters into equation (3.6), we rewrite the Laplace equation in its asymptotic form as

6.4 $$\frac{(P_{l}-P_{a})a}{\sigma }\approx \frac{e^{4}}{{\left(\theta^{2}+e^{4}\right)}^{3/2}}-\frac{1}{\left(R-1\right)},\;\theta \to 0,\;e\to 0,\;R>1.$$

When $\theta \approx \pm e^{4/3}{\left(R-1\right)}^{1/3}$, the solution of equation $(P_{l}-P_{a})a\text{/}\sigma \approx 0$ can be matched to the zero pressure at the washer sides. As one moves further towards parallel *B*, decreasing the angular coordinate *θ*, the pressure increases because of the first term in equation (6.4). In the vicinity of parallel *B*, the pressure becomes anomalously large, $(P_{l}-P_{a})a\text{/}\sigma \approx 1\text{/}e^{2}$. At parallel *D*, similar arguments suggest that the pressure remains anomalously large with a similar asymptotic behaviour $(P_{l}-P_{a})a\text{/}\sigma \approx 1\text{/}e^{2}$. Equation (6.4) accurately describes the pressure distribution near the edge (figure 11).

This analysis reveals that the attractors $\pm \theta_{c}$ must remain separated from the parallel *θ* = 0 where the pressure is expected to be high and the liquid will be squeezed out immediately towards the proboscis sides. The curvature associated with the coil radius *R* changes the scenario of a straight washer-like proboscis e→0, creating the two attractors in the vicinity of parallel *B* at *θ* = 0.

## 7. Pressure distribution in internal liquid films inside elliptical food canals of coiled proboscises

**Figure 12.**
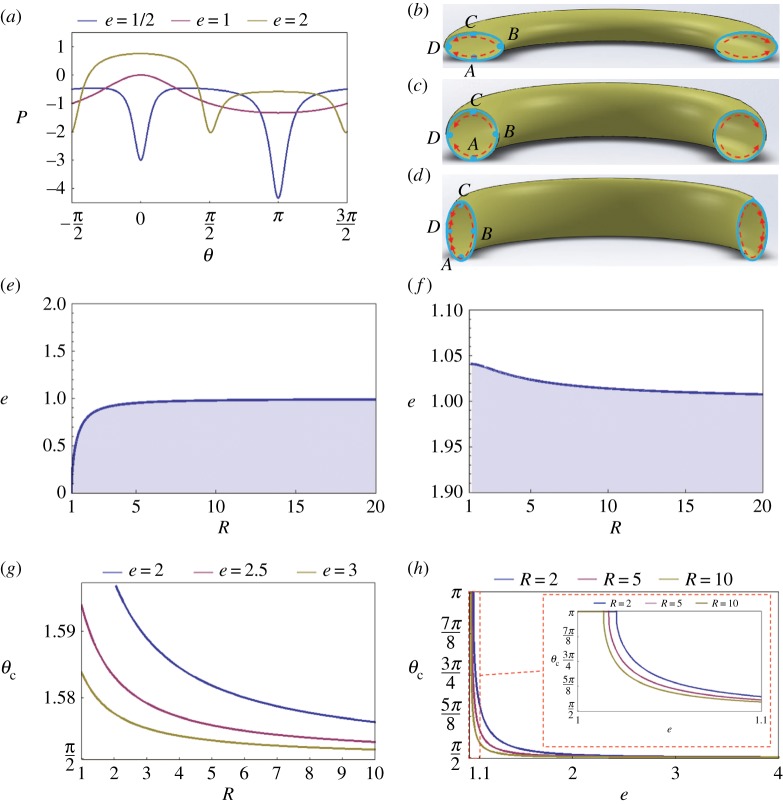
(*a*) Pressure distribution of a thin uniform liquid film deposited on the internal wall of a circular ring made of an elliptical tube with ring radius *R* = 2 at different cross-section ellipticities *e* = 1/2, 1 and 2. (*b*) Flow inside the internal liquid film when *e* = 1/2. (*c*) Flow inside the liquid film when *e* = 1. (*d*) Flow inside the liquid film when *e* = 2. (*e*) Diagram of transition from a single attractor at *θ* = 0 (shaded region) to two attractors (white region). (*f*) Diagram of transition from a single attractor at *θ* = *π* (shaded region) to two attractors (white region). (*g*) Influence of ring radius *r* on *θ*_c_ when the minimum pressure position is not at the critical positions *θ* = 0 and *θ* = *π*. (*h*) Influence of ring radius *e* on *θ*_c_ when the minimum pressure position is not at the critical positions *θ* = 0 and *θ* = *π*.

We relax the assumption about the circularity of the food canal and apply equations (2.1), (2.2) and (3.7) for the pressure distribution analysis. With the ideas of §6.2 in mind, the pressure distribution and associated scheme of fluid flow in the film are shown in figure 12*a*. Compared to circular food canals, in the case of washer-like food canals illustrated by the *e* = 1/2 curve in figure 12*a*, the pressure reaches its absolute minimum at the dorsal legular band *θ* = *π*; an additional local minimum of pressure occurs at the ventral legular band *θ* = 0. The pressure reaches its maximum at the two mirror-symmetrical positions near attractor *θ* = 0. Therefore, due to this spontaneously built differential of the capillary pressure, the film will move towards the parallels, *θ* = 0 and *θ* = *π*, where it will form a meniscus (figure 12*b*).

For proboscises with the food canal elongation perpendicular to the loop plane, *e* = 2 illustrates this case, the pressure reaches its absolute minimum at two mirror-symmetrical attractors specified by the angles $\pm \theta_{c}$. Thus, the film will move towards these parallels and will collect there as menisci (figure 12*d*).

Equations (6.2) and (6.3) can be used to identify the transition from a single attractor to two attractors. Indeed, equation (3.7) for the pressure distribution of the internal film differs from equation (3.6) for the external film only by the negative sign, which will not affect the inflection point conditions defined by equations (6.2) and (6.3). However, the meaning of each area in the diagram in figure 12*e,f* changes, compared with figure 10*e,f*.

The locations of the two parallel–attractors specified by the angles $\pm \theta_{c}$ are influenced by ring radius *R* and proboscis ellipticity *e* (figure 12*g,h*). At given ellipticity *e*, angle *θ*_c_ decreases along with ring radius *R* (figure 12*g*), approaching *θ *= *π*/2 as radius *R* goes to infinity and the proboscis straightens out. At given radius *R*, angle *θ*_c_ decreases along with ellipticity *e* (figure 12*h*), approaching *θ *= *π*/2 as ellipticity goes to infinity and the food canal is transformed into a coiled slit-like channel. Thus, menisci are formed at the ends of this slit-like channel, as expected.

## 8. Discussion

Liquid collection is a critical initial step in fluid feeding [47]. However, fluids available from floral corollas and other food sources are often limited. Fluid availability, therefore, can be a significant determinant of foraging behaviour [48]. Maximizing fluid acquisition and minimizing evaporation, thus, become essential for efficient feeding and water balance, and should be under strong selection pressure. Physical principles of the proboscis that enhance fluid collection would have been important in palaeoenvironments with limited availability of suitable resources, and might have been critical in the evolution of the proboscis [10]. Our findings suggest that coiling and bending have adaptive value beyond the conventional benefits of packaging and protecting the proboscis; these movements provide an additional means of optimizing fluid intake.

When a butterfly inserts its proboscis into a flower or applies it to a liquid pool or surface, fluid gathers on the proboscis as a film, similar to industrial dipcoating. Liquid evenly distributed on a straight proboscis must then be transferred to the legulae where the interlegular gaps will allow fluid to enter the food canal. Thus, the second step in fluid feeding involves directional movement of the liquid. Bending or flexing of the proboscis causes dewetting—movement of fluid to the legular bands of permeability. On a straight proboscis, the ventral and dorsal legular bands are equivalent attractors of fluid. Once the proboscis bends, the attractors are no longer symmetrical, and liquid moves to the dorsal legulae. Bending brings into play gravitational forces; any increase in gravity will aid directional movement of fluid. The process would be facilitated during feeding as the proboscis undergoes flexion at the knee-bend (*sensu* Krenn [1]) or assumes a J shape, as well as between feeding bouts when the insect bends or coils the proboscis as it moves between flowers. Passive fluid collection would be aided not only by gravity, but also by acceleration from the sweeping and mopping movements of the proboscis, as described [49] for fruit- and sap-feeding Lepidoptera. An important materials feature aiding fluid movement includes the chemical and physical composition of the proboscis surface. The fine surface sculpture of the proboscis typically has micro-ridges and valleys that aid capillarity and direct fluid towards the hydrophilic legulae [11].

Water conservation could be enhanced not only during actual feeding but also by efficiently recycling body fluids. Many Lepidoptera produce saliva or exude water droplets from the anus to solubilize nutrients and make them available for uptake via the proboscis. Hesperiids, for example, direct the abdomen anteriorly to exude droplets of fluid onto dry surfaces and then reimbibe the liquid [21], particularly on dry days (unpublished observations) when conservation of fluids is most critical. Recycling essential fluids in saliva and exudates could be facilitated by proboscis bending and coiling.

The dorsal and ventral legular bands of the linking mechanisms at the C-edges of the galeae are hydrophilic [11,41]. Thus, fluid readily spreads over the legular bands and sinks into the food canal through the interlegular gaps. The insect, therefore, is tasked to bring the liquid to the dorsal and ventral legular bands while drinking from floral corollas or other food sources. Our results corroborate our hypothesis that liquid is passively collected at the permeable dorsal and ventral legular bands due to proboscis ellipticity, as seen from the analysis of pressure distribution in a thin film covering an elliptical proboscis. An elliptical, rather than circular, proboscis brings the contact line of the meniscus higher on the drinking region, increasing the number of interlegular spaces covered with liquid [11].

This mechanism of nectar collection is important for Lepidoptera with long proboscises, such as *Xanthopan morganii praedicta* [44]. The moth inserts the proboscis into the flower and after about one second withdraws it. In so doing, the moth acquires a liquid film, and as it removes the proboscis from the corolla, the film remains on the exterior surface of the proboscis. According to our results, this film is unstable and is prone to move towards the dorsal and ventral legulae.

Proboscis ellipticity is more notable in non-flower visiting butterflies and moths that probe sap flows and animal wastes (e.g. dung) with bacteria and sticky compounds [1,50]. These insects move the liquid from the sides of the proboscis towards the central (legular) area, which is subject to rubbing during coiling and uncoiling of the proboscis such that the probability of removing debris is greater. Lepidoptera, thus, solve the problem of keeping their proboscises clean, while at the same time acquiring liquid food.

Figure 9*d* shows that the pressure differential between the dorsal and ventral legular bands increases when the radius of the proboscis coil, *R*, decreases. The drinking region with enlarged interlegular pores is near the proboscis tip. Therefore, by coiling the proboscis (figure 1*j*), the liquid moves faster under a stronger pressure differential. This same coiling scenario supports the movement of liquid from distant regions of the proboscis towards the drinking region; the pressure at the ventral band (absolute minimum in figure 9*a*) decreases as the insect bends its proboscis. Therefore, the 3–2–1 sequence of coiling (figure 1*j*) is important: these steps are natural and ensure that liquid will not be lost but will be collected at the ventral legular band of the outmost loop and then transferred to the more permeable dorsal legular band [10] of the neighbouring loop.

To quantitatively estimate the pressure differentials, we calculated capillary pressure for films on the proboscises of five species of butterflies, based on geometric parameters of their proboscises [11] (table 2). The pressure difference between the dorsal and ventral legular bands is small; it requires about a 2% pressure difference between the pressure differential forcing the liquid to move from the proboscis sides (parallels *A* and *C*) to the dorsal legular band (parallel *D*) when the loop radii are of the order of a millimetre. Thus, when these butterflies coil their proboscises, they trigger liquid collection by lateral movement of liquid films towards the drinking region.

**Table 2.** Capillary pressure in a thin liquid film coating the proboscis exterior. Atmospheric pressure is taken as the reference, *P*_a_ = 0. AM is the absolute minimum pressure at the ventral legular band (parallel *B* in figure 9); LM is the local minimum pressure at the dorsal legular band (parallel *D* in figure 9); DLA is the difference between LM and AM; Max is the maximum pressure at parallels *A* and *C* in figure 9; and DML is the difference between Max and LM. Proboscis cross section is assumed to be elliptical with semi-axes *O*_1_*D* = *a* and *O*_1_*C* = *b* (figure 2).

				pressure (Pa)				
species	*a* (μm)	*b* (μm)	*r* (mm)	AM	LM	DLA	Max	DML
*Danaus plexippus*	56	135	1.5	173	270	97	3125	2874
*Papilio glaucus*	65	182	1.5	92	189	97	3128	2939
*Vanessa cardui*	36	105	1.5	187	284	97	5882	5598
*Limenitis arthemis astyanax*	32	252	1.5	−13	84	97	17 866	17 782
*Polygonia interrogationis*	48	149	1.5	107	204	97	4695	4491

Greater proboscis ellipticity is associated with butterflies that routinely feed from wetted surfaces rather than from floral corollas. This ellipticity confers adaptive value when drinking from films because the contact line of the meniscus rises higher on the proboscis, compared with a cylindrical proboscis, thereby covering more interlegular spaces with fluid [11]. We found that capillary pressure suggests a relationship with feeding habits. The greatest pressures are associated with surface-film feeders (e.g. *Limenitis arthemis astyanax*) and the lowest pressures with routine flower visitors (e.g. *Danaus plexippus* and *Papilio glaucus*) (table 2). Thus, the extremes of this pressure range reflect two long-recognized feeding guilds of Lepidoptera: flower visitors (nectar feeders) and non-flower visitors (non-nectar feeders) [42].

**Figure 13.**
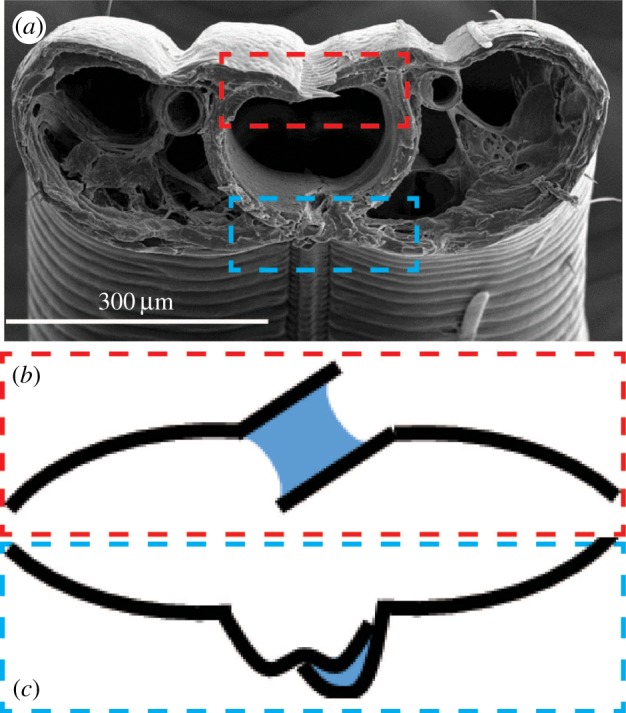
(*a*) Scanning electron microscope image of the proboscis of *Manduca sexta* in cross section, showing the position and structure of dorsal (red box) and ventral (blue box) legulae. Schematic showing capillary adhesion of (*b*) dorsal and (*c*) ventral legulae (black lines) by trapped fluid (blue).

Liquid collection at the dorsal legular band from inside the food canal (as suggested by the analysis summarized in figures 7 and 9) suggests that wetting of the dorsal and ventral legular bands benefits lepidopterans. An important implication of this effect is related to the mechanism of holding the paired galeae together, especially at the dorsal legular band where the legulae do not have a lock-and-key mechanical linkage, in contrast to the ventral band for which this mechanism was identified long ago [51–54]. The dorsal legulae weakly overlap one another, forming slit-like pores of hundreds of nanometres [10]. In the slit pores formed by two adjacent legulae, the liquid bridge could create a colossal capillary pressure, of the order of 10 atmospheres (figure 13). Because the legulae are hydrophilic, this capillary pressure tends to bring the legulae together and hold them in place, yet allow them to slide over one another with little resistance [17–20].

## 9. Engineering applications

**Figure 14.**
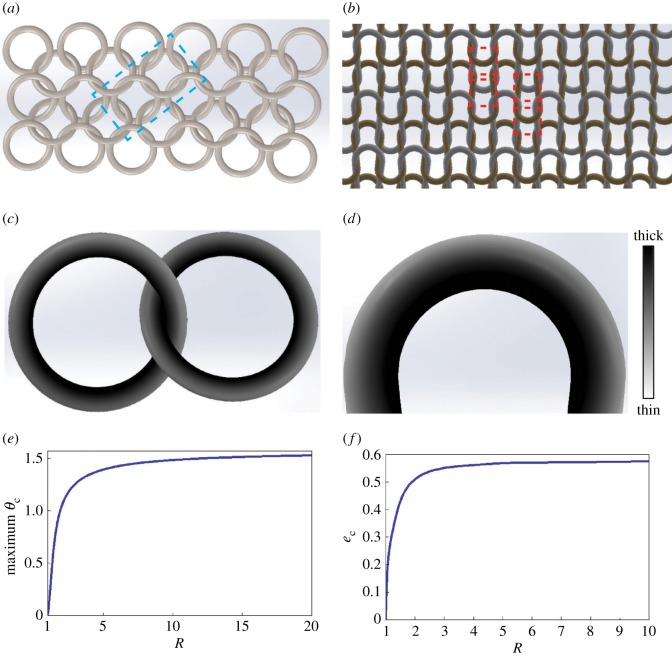
(*a*) Schematic of mail armour and (*c*) magnified picture of a ring couple with a non-uniform finish thickness. (*b*) Schematic of a knitted fabric. Some parts of the loop boxed by the dashed lines can be modelled in a first approximation by circular rings. (*c*) Illustration of the effect of non-uniform film thickness on mail armour rings: the darker, thicker films protect the rings against corrosion if water is trapped at the ring connections. (*d*) In the knitted fabric, the darker, thicker film is located at the loop interior while the brighter, thinner film is at the loop exterior. (*e*) Position of the finish collection as a function of radius *R* of a washer-like ring of ellipticity *e*_c_ specified in (*f*).

Analysis of the stability of liquid films on the external surface of the proboscis is useful for engineering applications involving an elliptical fibre bent into a loop. One example would be mail armour, which is usually made of metal rings (figure 14*a*). The main enemy of mail armour is corrosion, caused by water films that might be left on the ring surface after a rain. To prevent corrosion, water should collect at those sides of the ring where it can easily evaporate or be shaken off, that is, at the ring sides. According to the analysis in §6.2, the most attractive candidates for mail armour should have ellipticity less than one, *e* < 1 (figure 10*b*). In this case, one would intuitively think of using washer-like rings with *e* close to 0. These rings, with a high curvature at *θ* = 0, would force the liquid to flow towards the rings' flattened sides. However, the analysis summarized in figure 10*h* shows that the most attractive positions for water to flow are still near the ring edges where water can bridge two adjacent rings and might be trapped between them. The most distant position from the ring edges is determined by the maximum *θ*_c_ (figure 10*h*). In figure 14*e* these maximums are plotted as a function of ring radius *R*. In figure 14*f*, we plot the critical ellipticity *e*_c_ taken from figure 10*h*, corresponding to the given maximum *θ*_c_; this is another version of figure 14*e*, which is useful for ring design.

If the rings need to be coated with an anti-corrosion layer, our analysis suggests that elliptical rings would never have a uniform coating thickness (figure 10*a*). When a liquid film is deposited on a ring surface, the liquid will move towards the ring half closer to the axis of rotation. Therefore, the final coating layer will be thicker at the ring half closer to the axis of rotation (figure 14*c*). This effect is attractive for coating applications; it ensures that the thicker anti-corrosion coating will be deposited on its own at the ring contacts in the mail armour where water is likely to be trapped.

Another example is the design of knitted fabrics, in which the fibres or yarns are bent in loops forming the structure shown schematically in figure 14*b*. In a first approximation, these loops can be modelled as parts of a circular loop. Because the fibres and yarns are typically round in cross section, the results summarized in figure 10*c* can be applied to the analysis of behaviour of finishes or inks at the fibre or yarn level. By introducing a local system of coordinates placing its centre at the centre of each loop, one can directly use the results of §7. As follows from that analysis, liquid is expected to move towards the parallel *θ* = 0, as shown in figure 14*d*. This effect can be used, for example, in making a fabric with a gradient of coloration at the loop scale: the outermost layers of the loop will be less coloured than those sitting closer to the loop axis.

## 10. Conclusion

The capillary effects of a coating film inside or outside an elliptical hollow fibre were studied with regard to insect biology and applications to textile engineering. In thin films where the air–liquid interface is positioned almost parallel to the fibre surface, capillary pressure can be estimated by knowing the profile of the fibre surfaces supporting the films. Using this thin-film approximation, we examined the stability of liquid films on a hollow fibre with elliptical cross section coiled in a ring. The shape and movements of the proboscis represent evolutionary compromises between selection forces for optimal coiling, fluid uptake, and self-cleaning. Here we show that movements of the proboscis have heretofore unappreciated adaptive value in collecting fluid. Specifically, we show that coiling and bending of the proboscises of butterflies and moths facilitate fluid collection (table 3). The phenomenon of liquid collection by the proboscis and its facilitation via movement represents yet another deviation from the conventional drinking-straw model of the proboscis. Some possible applications to textile engineering where this effect of capillary instability of liquid films is important are discussed.

**Table 3.** Summary of steps for fluid collection by lepidopteran proboscises.

condition	cross section	activity	consequence
straight	circular	if proboscis length is greater than circumference, film forms droplets due to Plateau–Rayleigh instability	fluid is not necessarily collected at the legular bands
coiled	circular	external film moves to inner radius (figures 7*d,f* and 10*c*); internal film moves to outer radius of the food canal (figures 7*c*,*e* and 12*c*)	fluid moves to ventral legulae on the external surface of the proboscis, and to dorsal legulae in the food canal
straight	elliptical; dorsoventrally flattened (ribbon-like)	external film moves to flatter areas (figure 3*e*); internal film moves to more curved areas of the food canal (figure 3*f*)	fluid moves to dorsal and ventral legulae on the external surface of the proboscis, and away from dorsal and ventral legulae in the food canal
coiled	elliptical; dorsoventrally flattened (ribbon-like)	external film moves to inner radius (figure 10*d*); internal film moves to outer radius of the food canal (figure 12*d*)	fluid moves to ventral legulae on the external surface of the proboscis and to regions near dorsal legulae in the food canal
straight	elliptical; laterally flattened, legular bands are positioned at the most curved edges	external film moves to flatter areas (figure 3*e*); internal film moves to more curved areas of the food canal (figure 3*f*)	fluid moves away from dorsal and ventral legulae on the external surface of the proboscis, and to dorsal and ventral legulae in the food canal
coiled	elliptical; laterally flattened (washer-like)	external film moves to inner radius (figure 10*b*); internal film moves to outer radius of the food canal (figure 12*b*)	fluid moves to regions near ventral legulae on the external surface of the proboscis and to dorsal legulae in the food canal

## Supplementary Material

Pressure distribution analysis

## Supplementary Material

Critical pressure calculation

## Data accessibility

The Mathematica codes supporting this article have been uploaded as part of the electronic supplementary material.
